# Genetic dissection of seedling vigour in a diverse panel from the 3,000 Rice (*Oryza sativa* L.) Genome Project

**DOI:** 10.1038/s41598-019-41217-x

**Published:** 2019-03-18

**Authors:** Kai Chen, Qiang Zhang, Chun-Chao Wang, Zhi-Xia Liu, Yi-Jun Jiang, Lai-Yuan Zhai, Tian-Qing Zheng, Jian-Long Xu, Zhi-Kang Li

**Affiliations:** 1 Institute of Crop Sciences/National Key Facility for Crop Gene Resources and Genetic Improvement, Chinese Academy of Agricultural Sciences, Beijing, 100081 China; 2 Agricultural Genomics Institute at Shenzhen, Chinese Academy of Agricultural Sciences, Shenzhen, 518120 China; 3 Institute of Rice Research, Guangdong Academy of Agricultural Sciences, Guangzhou, 510640 China

## Abstract

Seedling vigour (SV) is important for direct seeding rice (*Oryza sativa* L.), especially in a paddy-direct seeding system, but the genetic mechanisms behind the related traits remain largely unknown. Here, we used 744 germplasms, having at least two subsets, for the detection of quantitative trait loci (QTLs) affecting the SV-related traits tiller number, plant height, and aboveground dry weight at three sampling stages, 27, 34, and 41 d after sowing. A joint map based on GAPIT and mrMLM produced a satisfying balance between type I and II errors. In total, 42 QTL regions, containing 18 (42.9%) previously reported overlapping QTL regions and 24 new ones, responsible for SV were detected throughout the genome. Four QTL regions, *qSV1a*, *qSV3e*, *qSV4c*, and *qSV7c*, were delimited and harboured quantitative trait nucleotides that are responsible for SV-related traits. Favourable haplotype mining for the candidate genes within these four regions, as well as the early SV gene *OsGA*2*0ox1*, was performed, and the favourable haplotypes were presented with donors from the 3,000 Rice Genome Project. This work provides new information and materials for the future molecular breeding of direct seeding rice, especially in paddy-direct seeding cultivation systems.

## Introduction

With economic and social development, cultivating systems for rice (*Oryza sativa* L.) production have changed. At present, direct seeding rice (DSR) has been widely adopted by farmers and is just as important as the traditional transplanting rice (TTR) system, which is now performed with the assistance of mechanical tools in China. In other parts of Asia, the adoption of DSR in place of TTR has also increased because of its labour and energy savings^1^. DSR can significantly reduce water consumption and labour requirements, while increasing system productivity and resource use efficiency^2^. Moreover, it can reduce greenhouse gas emissions during rice cultivation^3^.

In practice, there are at least two types of cultivating systems for DSR, paddy direct seeding (PDS) and upland direct seeding (UDS), also known as aerobic direct seeding. In UDS, the lack of a ‘head start’ and the absence of a standing water layer to suppress weeds make it highly vulnerable to weeds, causing severe yield losses compared with other rice ecosystems^4^. Thus, seedling vigour (SV), especially early SV (ESV), which refers to the SV for seedlings less than 28 d old, is imperative for crop stand establishment and weed competitiveness^4,5^. In PDS, however, the control of weeds is much easier owing to the standing water layer. Thus, the late SV (LSV), 28 d after sowing (DAS) is more important for the population type and final grain yield, especially in PDS. This is similar to the rice in the TTR cultivating system. Nevertheless, the growth of rice is different for seedlings under TTR and PDS conditions owing to the transplanting recovery procedure.

Substantial genetic variation in SV exists within the rice gene pool^6–8^. A lot of research has focused on SV, especially its molecular mapping. Many QTLs for SV in rice have been identified in bi-parental-derived populations by QTL analyses^9–15^. Dynamic and conditional QTL mapping have been used for the detection of SV-related QTLs^16,17^. However, most of the QTLs detected in these studies were located in relatively large regions and favourable allele/haplotype mining was not performed. The mining of the germplasm diversity for important SV-related QTLs is still limited.

Recently, natural populations, with advantages over the bi-parental populations, have been used for identifying QTLs of complex traits^18,19^. QTLs and favourable haplotypes affecting mesocotyl length were identified in a set of 621 rice accessions by GWAS, and the combinations of superior haplotypes of *OsML1* and *OsML*2 increase mesocotyl length by up to 4 cm^20^. Using general and mixed linear model approaches, 16 and 10 SSRs, respectively, were identified as significantly associated with ESV traits in 96 rice lines selected from a set of 629 rice accessions^21^. The 3,000 (3 K) Rice Genome Project has produced a highly diverse pool carrying favourable alleles for different traits^22,23^. Determining important loci with less background interference has been a challenge for GWAS. Additionally, widely used methods based on single marker analyses accumulate errors with the application of multiple tests. Thus, a multiple test correction, such a Bonferroni correction, is commonly required. A multi-locus random-single nucleotide polymorphism (SNP)-effect mixed linear model package, named mrMLM, was recently developed to overcome this shortcoming^24^.

Here, we adopted a traditional GAPIT method combined with mrMLM to dissect the SV variations in a sub-panel of 744 accessions from the 3 K rice genome. The aims of this study were to (1) identify QTL regions for SV at different DAS in diverse rice germplasms using GWAS, (2) to fine-map and mine favourable alleles for candidate genes in some important QTL regions, and (3) to compare the mapping efficacies and respective characteristics of the genomic association and prediction integrated tool (GAPIT) and mrMLM methods. This work could offer useful information for the molecular breeding of DSR, as well as being a reference for the joint use of GAPIT and mrMLM.

## Methods

### Plant materials and field experiments

A set of 744 germplasms was used in this work. Its members were randomly selected from the sequenced accessions of the 3 K Rice Genome Project^25^. The field work was conducted at the experimental station in Shenzhen (22.6°N, 114.4°E), Guangdong Province during the late season of 2017. Approximately 120 germinated seeds for each accession were directly sown with 20 cm between rows, 15 cm between individuals and two seeds per hill in the paddy plot. At the one-leaf stage, 60 similar seedlings for each accession, with a single seedling in each hill, were maintained. The extra seedlings were removed. The tests were arranged in randomised plots with three replications. Field management was carried out according to the local practice. Specifically, ~113 kg ha^−1^ of carbamide was applied at the 2.5-leaf stage to supply nitrogen. Butachlor was applied at ~1.8 kg ha^−1^ for weed control during paddy preparation before seeding, and it was applied again after the 3-leaf stage.

The first sampling (stage A) was carried out at 27 DAS, and the second (stage B) and third (stage C) samplings were carried out at 34 and 41 DAS, respectively. From each accession, 10 uniform individuals, except for those on the boarders, were evaluated for SV traits. The SV traits, tiller number (TN), plant height (PH), and aboveground dry weight (DW), were measured for each individual.

### Genotyping by sequencing and SNP extraction

The 744 accession panel was re-sequenced, with an average depth of more than 10×^25^. The cleaned reads were then mapped to the reference genome of ‘Nipponbare’ (IRGSP1.0), and ~14 M of high-quality SNPs were identified^25^. From these SNPs, a set of 2.9 M SNPs related to potential protein-coding areas was carefully selected. To build a SNP set for association studies, a subset of 27,921 SNPs was selected from the 2.9 M SNPs by choosing one SNP per 100 counts, as described in our previous GWAS mapping work^23^.

### QTL analysis, comparative mapping and haplotype analysis

Basic statistical analyses for SV traits were conducted with SAS^26^. For the graphing and plotting, both Excel and R scripts were adopted. The basic scenario of a compressed mixed linear model^27^ implemented in GAPIT^28^ was adopted for the association analysis between QTL-flanking markers and SV traits for the 744 sequenced accessions. The parameters for GAPIT were set in accordance with our previous report^23^. A relatively stringent threshold was adopted to identify significant correlations between the SNPs and SV traits with a −LOG_10_(P) value of 6.0. To minimise to the possibility of type II errors in QTL detection^29^, a relatively loose threshold of 3.0 was adopted for the QTL regions having supporting evidence from different traits or previous reports. The allelic effects were estimated by setting the Major.allele.zero = TRUE in GAPIT to identify the donors of favourable alleles and their effects on SV traits. We used the mrMLM package^24^ to confirm and complement the mapping results from GAPIT.

Comparative mapping was carried out against a reference sequence map, the GRAMENE annotation sequence map^30^, to compare the QTLs detected in this study with previously reported QTLs or genes known to be associated with SV-related traits in rice.

Favourable haplotypes for candidate genes were investigated jointly with the aid of Perl and R scripts as described in our previous reports^22,23^, with minor modifications. The procedure included the following six major steps: (1) Determine the sub-regions defined by quantitative trait nucleotides (QTNs) with supporting evidence from both GAPIT and mrMLM; (2) Fill in the sub-regions with more SNPs from the original 2.9 M sets^23^, and then carry out GWASs using mrMLM within these sub-regions; (3) Screen the key QTNs with evidences from different SV-related traits; (4) Search for candidate genes harbouring these key QTNs for SV traits; (5) Carry out haplotype analyses for these candidate genes for SV-related traits to confirm the GWAS mapping results; and 6) Determine the top donors carrying favourable haplotypes.

## Results

### Performances of SV-related traits in the sequenced accessions

**Figure 1:**
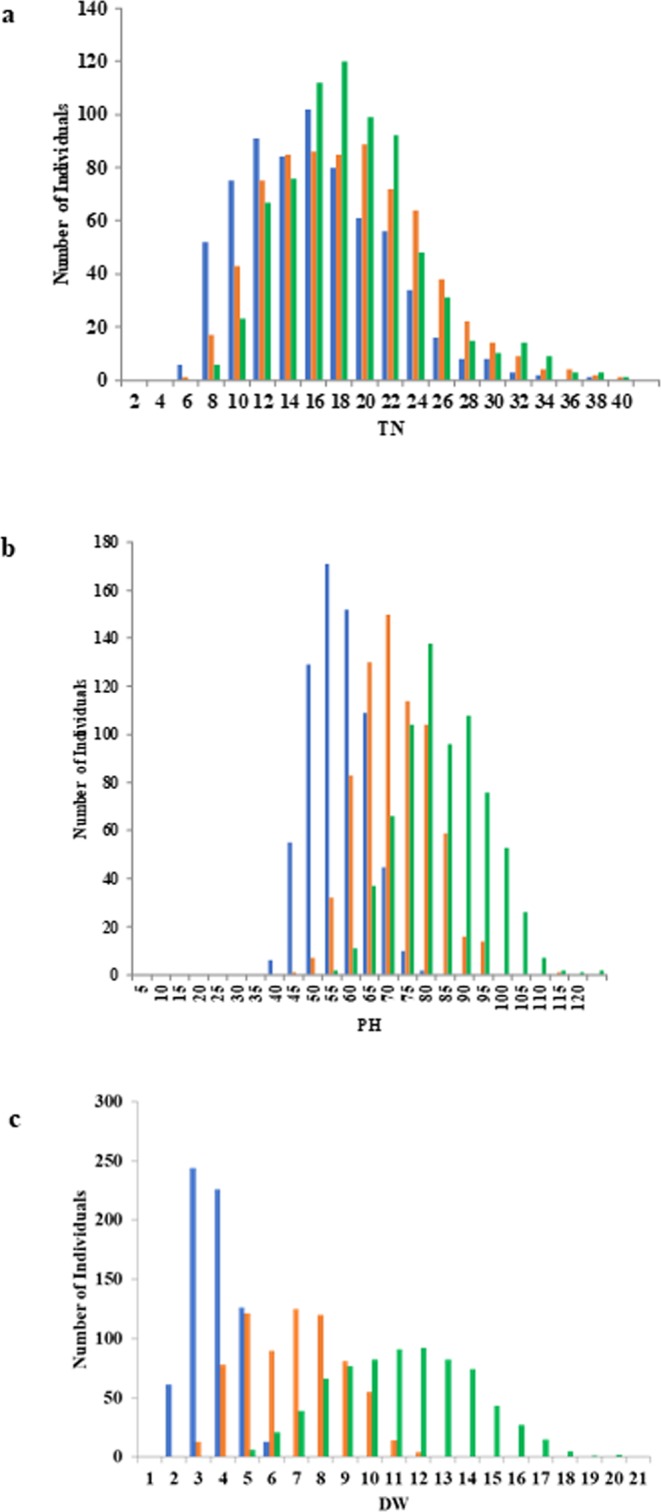
Distribution of three different traits at three stages of the 744 3 K-germplasms. (**a**) Tiller number (TN). (**b**) Plant height (PH). (**c**) Dry weight (DW). Three different colors represent sampling at 27 (blue), 34 (orange), and 41 (green) days after seeding.

As shown in Fig. 1a–c, the three measured major SV-related traits, TN, PH, and DW, were different at the three sampling stages (27, 34, and 41 DAS). For the three sampling stages, the TN had ranges of 2.4–34.2, 4–36.6, and 4.6–37.8, respectively, with mean values of 13.1, 15.6, and 16.0, respectively. For the three sampling stages, the PH had ranges of 30.3–71.8 cm, 38.2–105.2 cm, and 45.2–118.8 cm, respectively, with mean values of 49.8, 64.2, and 76.4 cm, respectively. For the three sampling stages, the DW had ranges of 0.39–4.92 g, 1.47–10.34 g, and 3.34–18.38 g, respectively, with mean values of 2.20 g, 5.37 g, and 9.77 g, respectively. The ranges of the phenotypic distributions became wider at each progressive sampling stage. One unique trait was DW, for which the distribution range increased significantly from sampling stages A to C (Fig. 1c). However, the distribution range of TN and PH almost remained almost unchanged from sampling stages A to C. The pattern change for PH based on the sampling stages was between those of the previous two traits.

**Figure 2:**
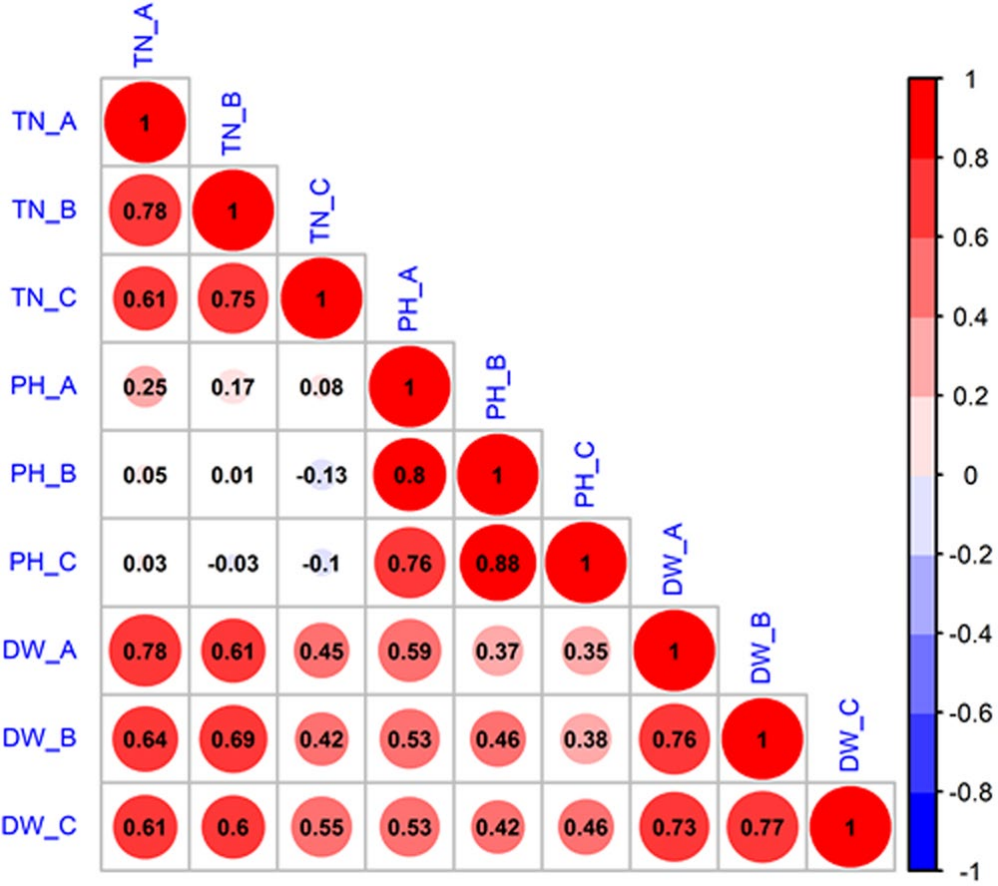
Correlations between three SV traits (TN = tiller number, PH = plant height, and DW = dry weight) at three stages (A = 27 DAS, B = 34 DAS, and C = 41 DAS) of the 744 3 K-germplasms. (1) Red = positive correlation, and blue = negative correlation; (2) the size of circle indicated the significance of correlations.

For each trait, all of the values from the different sampling stages were highly significantly correlated except for the correlations between PH and TN, and all of the correlations were in positive direction (Fig. 2). Especially, DW and TN were highly correlated having correlation coefficients greater than 0.60 at the first two sampling stages (27 and 34 DAS), but only ranged by 0.42–0.55 at the third sampling stage (41 DAS). However, the correlations between DW and PH (ranged by 0.35–0.59) were not as significant as the others.

**Figure 3:**
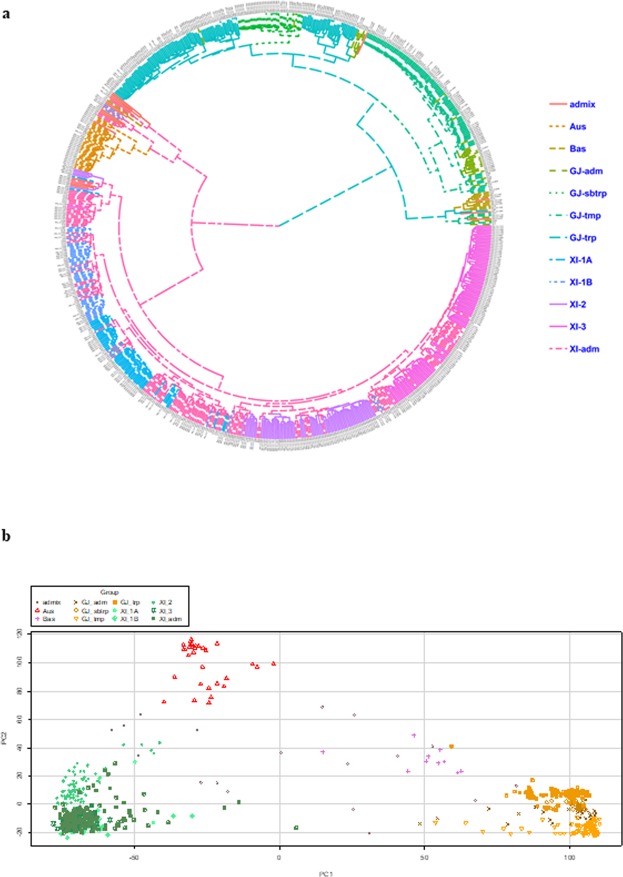
Phylo-tree (**a**) and PCA plots (**b**) based on the SNP genotyping data of the 744 germplasms.

Using the 27 K SNPs, sample clustering and a PCA were also performed. The PCA results for the 744 panel are shown in Fig. 3a, and the kinship between the 744 accessions is presented in Fig. 3b. Based on the PCA and the kinship analysis, the 744 accessions were divided into at least two large groups. Within each group, there were at least one or two subgroups. This was consistent with the two sub-species groupings of the rice accessions.

Linkage disequilibrium (LD) decay values were calculated throughout the genome, and the average values are shown in Supplementary Fig. 1. On average, LD blocks in this rice population of 744 germplasms extend up to ~50 kb.

### Identification of QTL regions controlling SV-related traits at three sampling stages

A total of 42 QTL regions (Table 1) were detected by GWAS mapping of the three SV-related traits at three sampling stages in 744 sequenced rice accessions. Among them, 28 QTL regions were responsible for TN. Among these, 15, 11, and 15 QTL regions were detected by GAPIT and 10, 7, and 12 QTL regions were detected by mrMLM at 27, 34, and 41 DAS, respectively. The averaged −LOG_10_(P) values for SV-related QTL regions affecting TN at 27, 34, and 41 DAS were 3.5, 3.5, and 3.7, respectively, with ranges of 3.0–4.4, 3.0–6.0, and 3.0–5.7, respectively, as determined by GAPIT/mrMLM), and 3.6, 3.6, and 3.7, respectively, with ranges of 3.1–4.7, 3.0–6.0, and 3.0–5.7, respectively, as determined by mrMLM. Three QTLs (*qSV2c*, *qSV5a*, and *qSV9b*) were active at all three sampling stages.

**Table 1:** Loci detected for seedling vigor (SV) traits.

Loci	Ch	Physical Range (bp)	Tiller number^b^			Plant height (cm)			Aboveground dry weight (g)			Reference^c^
			A^a^	B	C	A	B	C	A	B	C	
*qSV1a*	1	247,189–3,653,960	3.7(0.93)/3.4(−0.86)	3.0(0.91)/			3.3(1.91)/3.2(1.80)	3.3(2.34)/			3.1(0.48)/3.0(−0.45)	
*qSV1b*	1	16,208,209–18,798,435		3.4(2.44)/			3.4(−3.39)/				3.5(−0.78)/3.3(−0.75)	
*qSV1c*	1	22,738,546–27,100,563				3.4(1.50)/	3.2(2.73)/	4.6(−2.03)/4.9(1.94)	3.7(0.21)/3.7(−0.21)			*qGR-1* ^38^
*qSV1d*	1	32,045,736–38,610,998				5.2(1.85)/4.7(1.50)	3.4(1.70)/	4.5(2.78)/3.7(−2.35)	3.6(0.17)/3.2(−0.15)			*qSSL1b*^33^; *qRL-1*^34^; *qLA-1*^35^
*qSV1e*	1	41,726,360–43,170,168	3.5(0.95)/3.2(−0.84)		3.1(−1.18)/	3.0(1.01)/	3.8(2.20)/3.3(−1.96)		3.2(0.12)/			
*qSV2a*	2	130,359–2,619,648		3.2(1.12)/3.0(−1.03)		3.6(−1.15)/3.2(1.13)		3.1(−2.69)/		4.0(−0.46)/4.0(0.43)		*qFML2-1*^20^; *qRL-2*^35^
*qSV2b*	2	8,441,087–8,548,339	4.1(−0.97)/4.1(1.10)						3.6(−0.17)/3.4(0.16)			
*qSV2c*	2	14,150,759–32,211,753	3.1(0.72)/	3.0(0.99)/	3.4(1.74)/3.2(−1.59)	3.8(1.70)/3.2(−0.94)	4.6(2.23)/3.5(2.02)	4.7(2.66)/3.7(3.52)	3.3(−0.21)/3.2(−0.20)	3.0(0.26)/	3.0(−0.68)/	*qGR2*^31^; *qSEV-2-2*^16^ *qCSH2*, *qSDW2*^32^
*qSV3a*	3	1,249,933–3,314,418				3.9(−1.35)/3.2(1.27)			4.8(0.15)/3.2(−0.23)			
*qSV3b*	3	7,403,648–9,937,979						3.2(2.46)/	3.0(−0.33)/	4.8(0.29)/4.8(0.40)		*qFV-3-1* ^15^
*qSV3c*	3	13,807,940–17,181,394					3.7(3.23)/3.2(3.27)	3.3(2.99)/		4.4(0.32)/4.3(0.30)		
*qSV3d*	3	25,356,061–27,968,077		3.7(−0.75)/3.5(0.77)	3.3(1.80)/3.0(−1.66)		3.0(−2.14)/					*qSEV-3-3* ^16^
*qSV3e*	3	33,321,103–36,187,187			4.9(1.88)/5.0(−1.80)	6.1(−2.39)/6.9(−2.42)	4.1(3.75)/4.0(−1.80)					*qPHS3-2*/*OsGA20ox1*^36^; *qSEV-3-4*^16^ *qSV-3-2*^13^
*qSV4a*	4	1,218,150–14,879,283			3.6(1.33)/3.4(−1.23)	5.5(1.74)/5.2(2.02)	5.9(1.95)/5.2(2.18)	4.9(2.46)/4.7(2.93)	3.2(0.14)/	3.7(0.63)/		
*qSV4b*	4	22,422,275–31,068,550			4.4(−1.17)/4.3(1.07)			4.0(2.51)/3.2(−2.20)	3.2(0.16)/		3.5(1.30)/	*qSDW4*.*2*^37^; *qGP-4*^38^
*qSV4c*	4	34,839,052–34,912,830					3.4(−1.83)/3.2(1.70)					*qFML4-1* ^20^
*qSV5a*	5	465,348–940,789	3.0(1.32)/	3.6(−1.16)/3.4(−1.08)	3.7(−1.25)/3.7(−1.20)	3.1(−1.80)/3.0(1.69)						
*qSV5b*	5	4,776,365–8,393,651	3.1(−0.76)				3.9(1.43)/3.2(−1.25)	3.4(1.73)/	3.1(0.37)/			
*qSV5c*	5	17,838,397–20,584,909				6.2(−2.40)/5.9(−2.18)	4.8(−2.48)/4.0(−2.29)	4.4(−3.24)/4.1(−2.86)				*qFV-5-2*^15^/*qSEV-5-2*^16^
*qSV5d*	5	24,408,722–25,402,737		3.3(2.04)/3.2(−1.88)	3.8(−2.11)/3.7(1.98)							
*qSV6a*	6	5,533,676–9,671,184	3.2(0.88)/			3.9(−1.20)/3.2(−1.87)	3.2(−1.75)/	3.6(2.24)/3.0(−2.01)				
*qSV6b*	6	16,028,185–26,071,313	3.4(1.11)/3.2(−0.82)		3.1(1.20)/	6.7(1.79)/	4.4(2.12)/3.2(−1.87)	6.0(2.99)/5.5(−2.10)		4.2(0.45)/4.2(−0.26)	3.6(0.63)/3.4(−0.42)	*qDW-6*^34^; *qLDW-6*^35^
*qSV7a*	7	1,762,263–1,762,263				3.0(−1.43)/	4.4(−2.42)/3.7(−2.07)	3.1(−2.36)/				
*qSV7b*	7	5,264,789–12,326,358					3.7(4.57)/3.3(−1.57)	3.2(2.85)/	3.2(−0.18)/			
*qSV7c*	7	21,348,611–28,256,825	3.1(1.41)/3.2(−1.33)		3.3(1.37)/3.1(−1.25)	4.1(1.65)/3.4(−1.40)	4.3(2.27)/3.3(−1.86)	4.1(2.64)/3.2(−2.16)		3.0(0.38)/		*qSDW5-1*^11^; *qSL-7*^35^
*qSV8a*	8	3,087,823–4,401,568			3.1(1.69)/3.0(−1.55)	4.7(−1.33)/4.1(−1.18)	4.1(−1.70)/3.2(−1.40)			3.2(0.44)/3.1(−0.40)		
*qSV8b*	8	9,111,126–10,698,322				3.1(−1.10)/	3.3(−2.30)/3.0(−2.17)					
*qSV8c*	8	19,364,992–20,536,688			3.0(−1.39)/	3.3(−1.70)/	3.6(−1.70)/	4.3(2.80)/3.3(1.96)	4.4(−0.35)/			
*qSV8d*	8	24,092,918–25,738,117		3.1(0.81)/	3.5(0.90)/3.3(−0.84)				5.5(0.21)/5.2(−0.13)	3.1(0.27)/		*qSV-8-2*^13^; *qPR-8*^35^
*qSV9a*	9	245,065–851,691				3.4(−1.74)/				4.3(−0.41)/4.2(−0.74)		*qFML9-1* ^20^
*qSV9b*	9	3,948,813–4,258,518	3.6(−2.06)/3.7(−1.99)	6.0(−1.36)/6.0(−1.25)	5.7(−2.52)/5.7(−2.35)					3.7(−0.65)/3.5(−0.67)		
*qSV9c*	9	7,927,435–19,840,140		3.2(1.04)/3.0(−0.88)			4.4(3.42)/4.0(3.12)	3.2(2.68)/	4.6(0.18)/4.5(−0.12)		4.0(0.85)/4.0(−0.40)	*qLA-9* ^35^
*qSV10a*	10	4,602,477–4,690,337	4.4(−0.75)/4.7(1.52)						3.1(−0.12)/			
*qSV10b*	10	11,319,741–15,974,000	3.1(−0.88)/		3.1(0.94)/	3.7(−2.18)/	6.0(1.90)/5.0(−1.73)	4.5(−3.95)/3.5(3.73)				*qRDW10-1* ^11^
*qSV10c*	10	22,274,976–22,791,107	3.1(−0.81)/	3.3(−0.97)/3.4(1.83)					3.7(0.13)/3.2(−0.12)			*qFV-10* ^15^
*qSV11a*	11	1,856,603–6,602,981	4.1(1.51)/4.1(−1.46)			3.3(−1.23)/3.2(1.14)					4.9(0.56)/4.8(−0.41)	*qFML11-1*^20^; *qLN-11*^35^
*qSV11b*	11	14,276,914–17,001,696	3.3(−1.66)/				3.1(−1.88)/	3.2(2.47)/	3.5(0.15)/3.3(0.15)			
*qSV11c*	11	20,803,665–24,893,773					3.8(2.79)/3.5(−1.22)	3.0(2.41)/				
*qSV12a*	12	3,066,169–3,066,169			3.2(−2.20)						3.0(−0.95)	
*qSV12b*	12	18,299,090–18,299,090								3.6(−0.32)/3.5(0.29)		
*qSV12c*	12	23,456,727–23,737,117					3.2(−3.08)/				3.6(0.53)/3.5(−0.49)	
*qSV12d*	12	27,405,080–27,405,080	3.6(−1.59)/3.4(−1.49)						3.6(−0.24)/3.5(−0.22)			

^a^A, B, C = Sampling on 27, 34, and 41 days after seeding, respectively.

^b^The values of -Log10(P) (outside brackets) and allelic effects (in brackets) shown in front of the “/” were estimated by GAPIT, while those behind the “/” were estimated by mrMLM.

^c^References for nearby loci reported.

A total of 35 SV-related QTL regions were responsible for PH. Among these, at 27, 34, and 41 DAS, 20, 26 and 21, respectively, were detected by GAPIT and 12, 18, and 11 respectively were detected by mrMLM. Eleven QTL regions (*qSV1c*, *qSV1d*, *qSV2c*, *qSV4a*, *qSV5c*, *qSV6a*, *qSV6b*, *qSV7a*, *qSV7c*, *qSV8c*, and *qSV10b*) were active throughout all three sampling stages as assessed by GAPIT. Four of them (*qSV2c*, *qSV4a*, *qSV5c*, and *qSV7c*) were confirmed by mrMLM. The averaged −LOG_10_(P) values for SV-related QTL regions affecting PH at 27, 34, and 41 DAS were 4.2, 3.9, and 3.9, respectively, according to GAPIT, with ranges of 3.0–6.7, 3.0–6.0, and 3.0–6.0, respectively, and 4.1, 3.6, and 3.9, respectively, according to mrMLM, with ranges of 3.0–6.9, 3.0–5.2, and 3.0–5.5, respectively.

Of the 31 SV-related QTL regions responsible for DW, 18, 12, and 8, as assessed by GAPIT, and 10, 8, and 7, as assessed by mrMLM, were detected at 27, 34, and 41 DAS, respectively. Only *qSV2c* was active throughout all three sampling stages according to GAPIT and it was confirmed by mrMLM at 27 and 34 DAS. The averaged −LOG_10_(P) values for SV-related QTL regions affecting DW at 27, 34, and 41 DAS, were 4.2, 4.0, and 3.9, respectively, with ranges of 3.0–6.7, 3.0–6.0, and 3.0–6.0, respectively, GAPIT, and 4.1, 3.6, and 3.9, respectively, with ranges of 3.0–6.9, 3.0–5.2, and 3.0–5.5, respectively, according to mrMLM.

Approximately 11.5% of the SV-related QTL regions were responsible for TN throughout all three sampling stages (27, 34, and 41 DAS). When we analysed PH with a higher heritability, the percentage of stable QTL regions reached 33.3%; however, the DW value dramatically decreased to 3.3%

### Mapping results from GAPIT and mrMLM

As shown in Table 2, for the 28 SV-related QTL regions responsible for TN, GAPIT detected 26 (15, 11, and 15 for the three sampling stages, respectively), while mrMLM detected 14 (10, 7, and 12 for the three sampling stages, respectively). In a comparison between the two methods, 12 QTL regions overlapped (9, 7, and 11 for the three sampling stages, respectively). For the 33 QTL regions responsible for PH, GAPIT detected all of them, with 20, 26, and 21 for the three sampling stages, respectively, while mrMLM detected 27, with 12, 18, and 11 for the three sampling stages, respectively. The overlapping QTL regions between the two methods at the three sampling stages were 12, 18, and 11, respectively. For the 31 SV-related QTL regions responsible for DW, GAPIT detected 30, with 18, 12, and 8 for 27, 34, and 41 DAS, respectively, while mrMLM detected 23, with 10, 8, and 7 for the three sampling stages, respectively. The overlapping QTL regions between the two methods were 10, 8, and 6 at the three sampling stages, respectively.

**Table 2:** Comparisons of QTL region/QTN for seedling vigor detected by two different methods (GAPIT and mrMLM).

Trait^a^	Stage^b^	SNP_based			Loci_based		
		GAPIT	mrMLM	Overlap	GAPIT	mrMLM	Overlap
TN	A	23	13	12	15	10	9
	B	20	14	14	11	7	7
	C	22	16	15	15	12	11
PH	A	68	31	31	20	12	12
	B	82	39	37	26	18	18
	C	60	24	24	21	11	11
DW	A	61	33	33	18	10	10
	B	19	12	12	12	8	8
	C	28	16	15	8	7	6
Total Subtotal^c^		383	198	193	41	42	41

^a^TN = Tiller number, PH = Plant height, and DW = dry weight, respectively.

^b^A, B, C = Sampling on 27, 34, and 41 days after seeding, respectively.

^c^The number of loci excludes the duplicated counts of loci by different traits at different stages.

As shown in Supplementary Figs 2–4, Manhattan plots with similar patterns were obtained for both GAPIT and mrMLM. However, using the threshold settings, relatively fewer QTL regions with clearer backgrounds were obtained by mrMLM.

### Haplotype analysis of candidate genes for QTL regions

**Figure 4:**
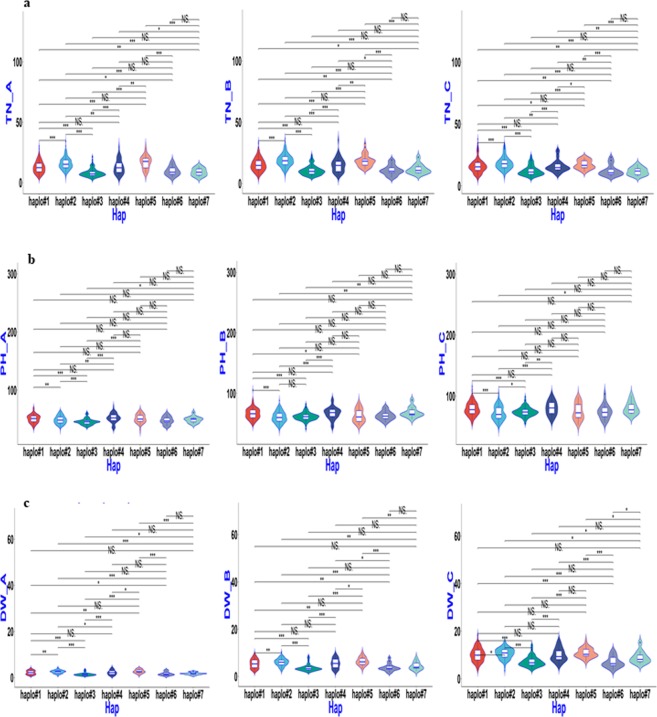
Boxplot for SV phenotypic values of two haplotypes of *Os01g0166800* (*qSV1a*) in the 744 germplasms. (**a**–**c**) TN, PH, and DW values for the three sampling stages 27, 34, and 41 DAS (left to right).

**Figure 5:**
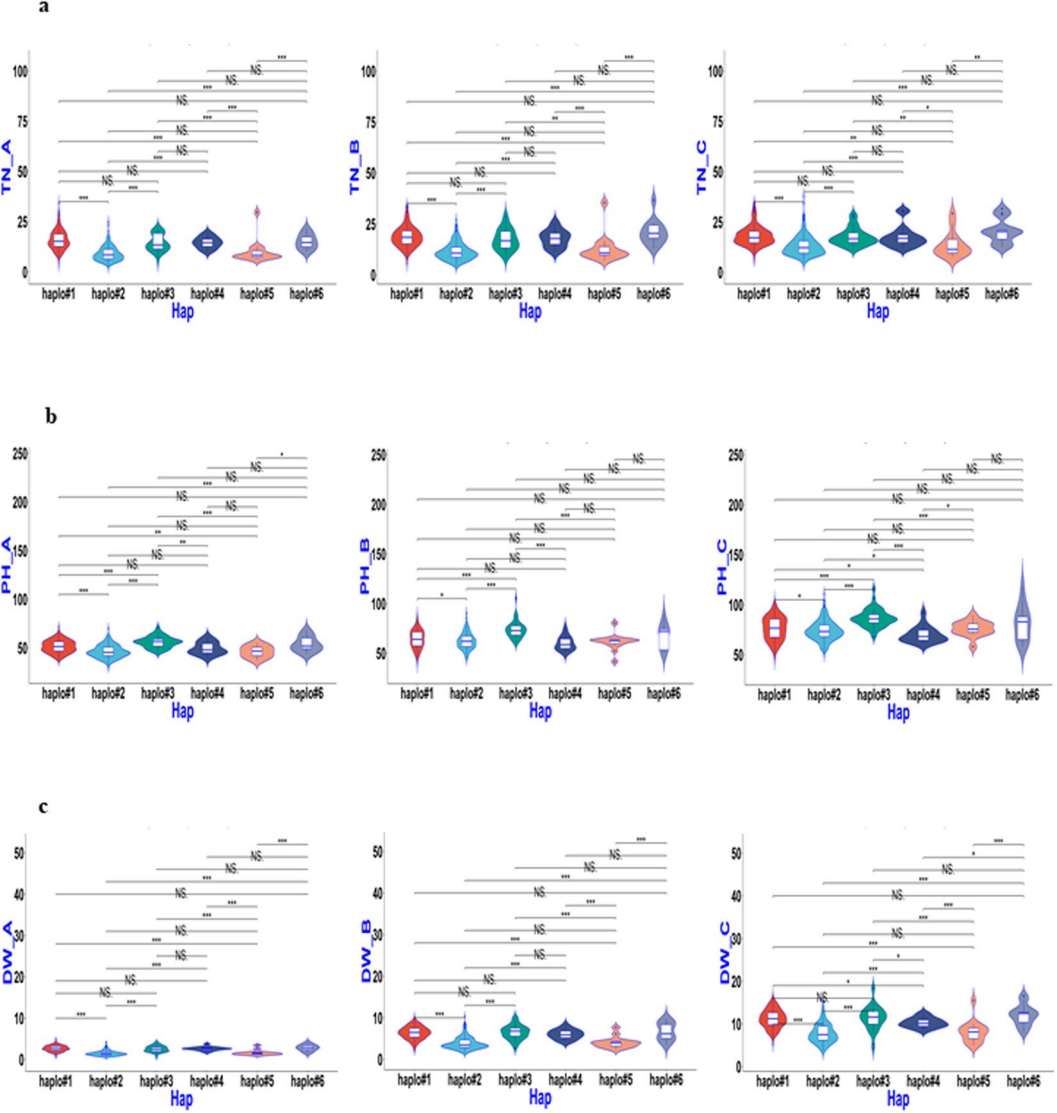
Boxplot for SV phenotypic values of two haplotypes of *Os03g0799700* (*qSV3e*) in the 744 germplasms. (**a**–**c**) TN, PH, and DW values for the three sampling stages 27, 34, and 41 DAS (left to right).

**Figure 6:**
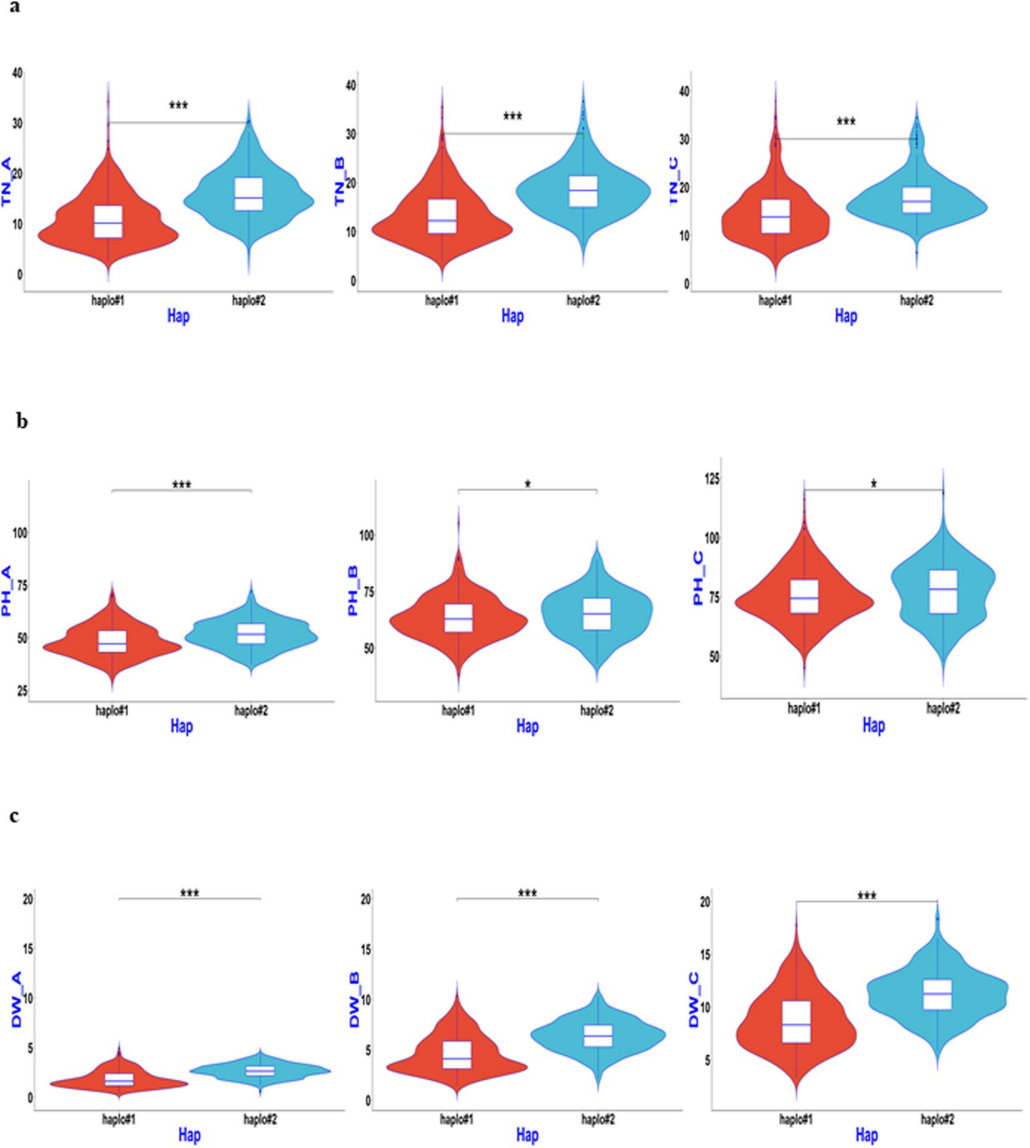
Boxplot for SV phenotypic values of two haplotypes of *OsGA20ox1* in the 744 germplasms. **(a**–**c**) TN, PH, and DW values for the three sampling stages 27, 34, and 41 DAS (left to right).

**Figure 7:**
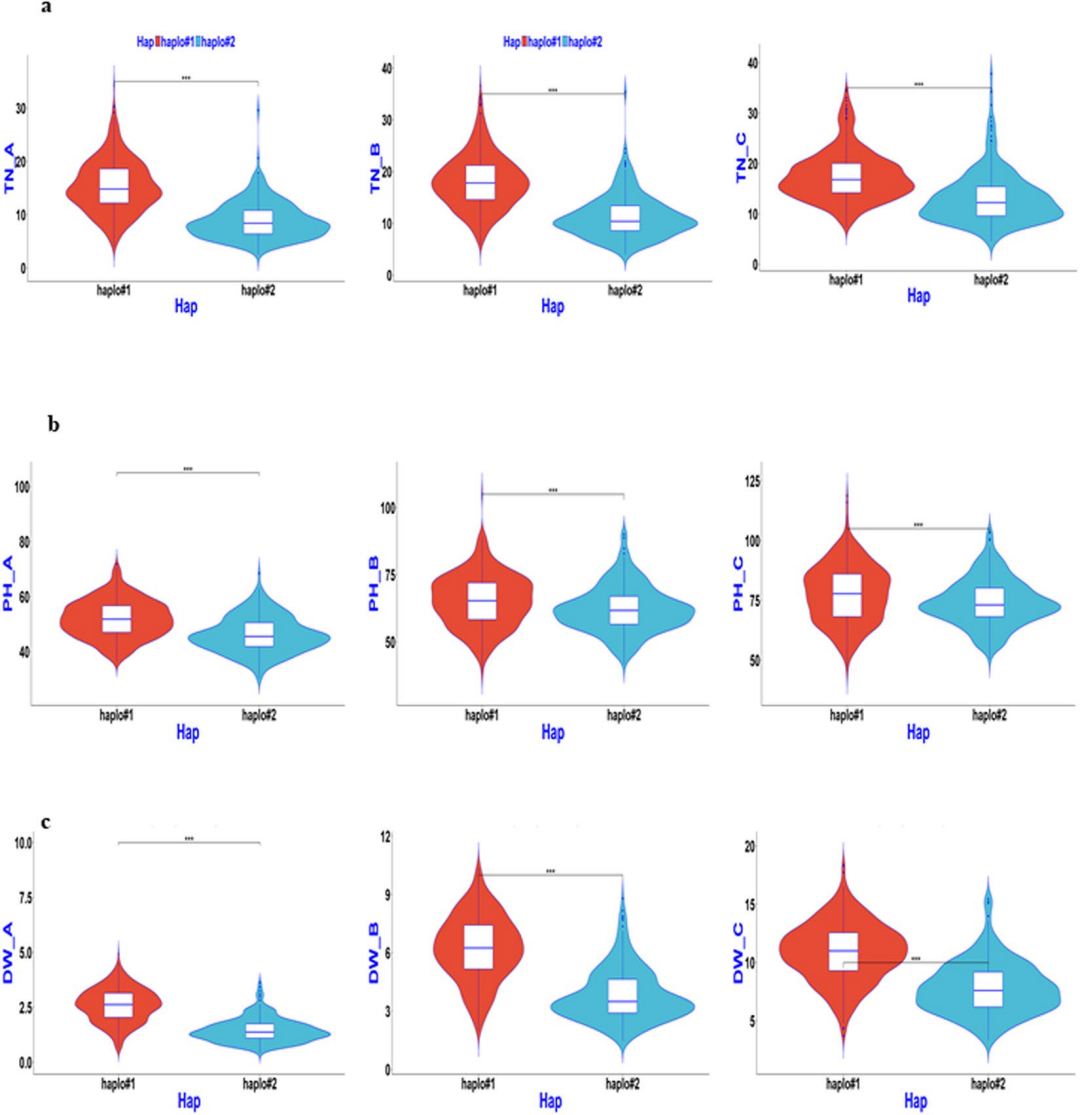
Boxplot for SV phenotypic values of two haplotypes of *Os04g0683600* (*qSV4c*) in the 744 germplasms. (**a**–**c**) TN, PH, and DW values for the three sampling stages 27, 34, and 41 DAS (left to right).

**Figure 8:**
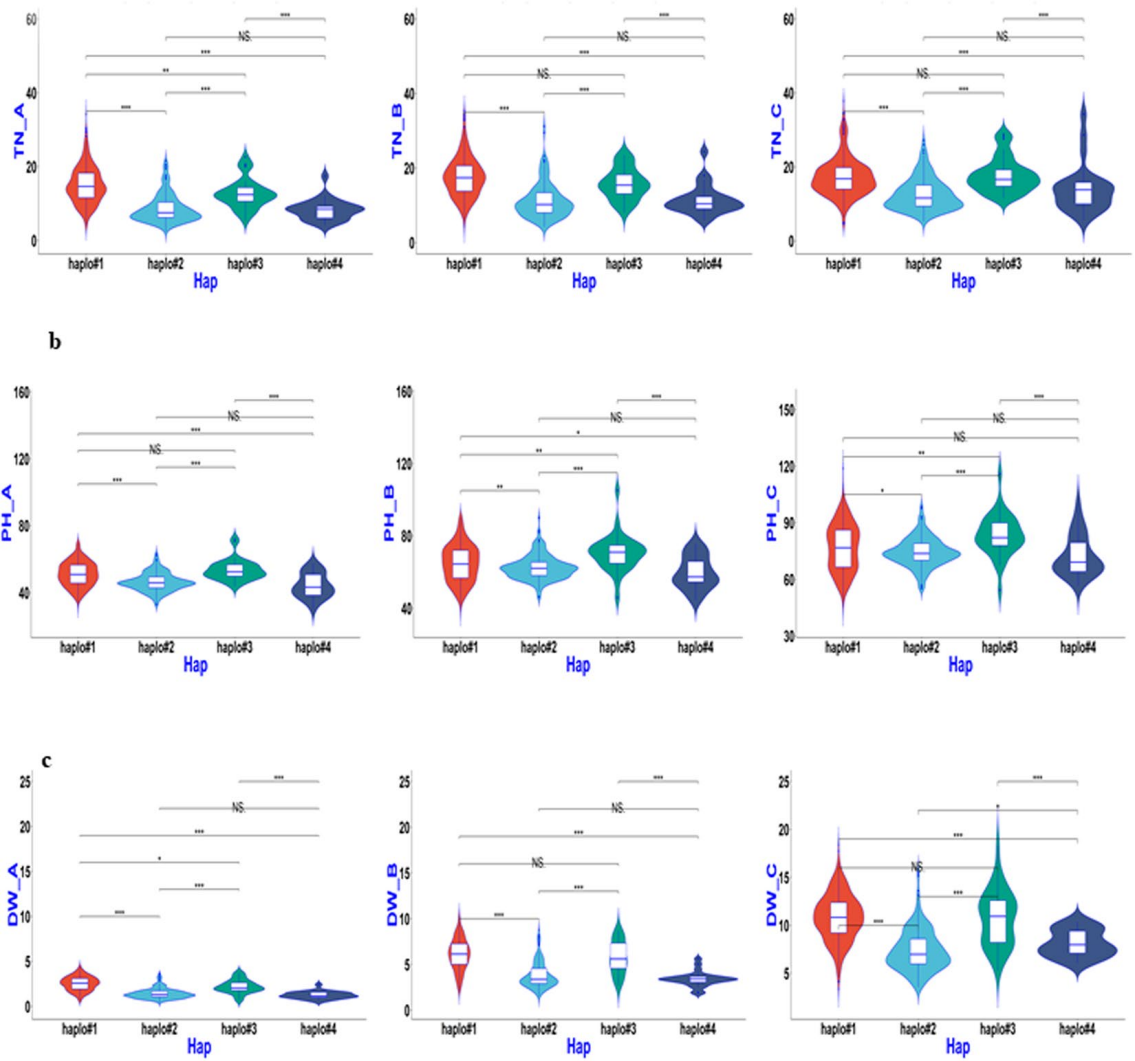
Boxplot for SV phenotypic values of two haplotypes of *Os07g0600400* (*qSV7c*) in the 744 germplasms. (**a**–**c**) TN, PH, and DW values for the three sampling stages 27, 34, and 41 DAS (left to right).

Based on the above association mapping results, four QTL regions, *qSV1a*, *qSV3e*, *qSV4c*, and *qSV7c*, were selected for fine-mapping using more SNPs. The region harbouring *qSV3e* was split into two sub-regions, which contained candidate *Os03g0799700*, with multi-evidenced QTNs, and *Os03g0856700*, with a single SNP but large −LOG_10_(P) value of 13.0 based on the ‘Nipponbare’ reference genome (Supplementary Table). Similarly, the other three QTL regions were delimited and harboured multi-evidenced QTNs after a sub-region analysis. Candidate genes containing significant SNPs or exactly located SNPs having phenotypic haplotype effects, as well as their donors, were analysed and are shown in Table 3 and Figs 4–8. *Os01g0166800*, *OS03g0799700* and *Os03g0856700* (*OsGA20ox1*), *Os04g0683600*, and *Os07g0600400* were determined to be candidate genes for *qSV1a*, *qSV3e*, *qSV4c*, and *qSV7c*, respectively, owing to significant differences in related traits among their different haplotypes. As shown in Fig. 4, haplotype 2 of *Os01g0166800* appeared to be elite, affecting both TN and DW without affecting PH, while haplotype 5 affected all three traits. Haplotypes 1 and 6 of *OS03g0799700* were recommended (Fig. 5), affecting TN and DW without significantly increasing PH, while haplotype 3 influenced all three traits. Two haplotypes of *Os03g0856700* (*OsGA20ox1*) appeared in the 744 germplasms (Fig. 6). Phenotypic differences among the three trait values (TN, PH, and DW) between the two haplotypes were highly significant at all three sampling stages, except for marginal differences in PH at 34 and 41 DAS (Fig. 6b). Haplotype 2 behaved better than haplotype 1 for *Os03g0856700*. For *qSV4c*, haplotype 1 was better than haplotype 2, while for *qSV7c*, haplotypes 1 and 3 were better than haplotype 2. The favourable haplotypes of *qSV4c* and *qSV7c* affected all the three SV-related traits (Figs 6 and 7).

**Table 3:** Favorable haplotypes and elite donors of candidate genes for four SV loci.

Loci	Candidate gene	Haplotype	TN^a^			PH			DW		
			A^b^	B	C	A	B	C	A	B	C
*qSV1a*	*Os01g0166800*	haplo#5	**11516**(22.2)^c^; 8645(23.4); **CX26**(29.2); CX240(21.8); CX377(27.4)	11693(26.4); **11516**(26.2); 12081(29.6); 9023(24.6); **CX26**(32.8)	11693(22.6); **11516**(22.8); 10824(21.8); 12081(23.0); **CX26**(23.0); CX377(21.8)	**11693**(64.0); 10695(64.6); 11849(60.6); 8697(61.6); 9160(60.6)	**11693**(85.1); 11802(82.8); 11643(78.7); 11849(80.2); 9472(76.8)	**11693**(95.8); 11145(96.2); 10695(99.0); 9472(93.2); 9160(94.9)	11693(4.0); 11920(3.8); **10824**(3.7); 10695(4.2); 8697(3.8)	11693(10.3); 11802(9.8); 11416(8.8); **10824**(9.1); 9472(8.3)	11920(13.5); 11416(13.1); **10824**(16.2); 9160(13.5); CX162(14.6)
		haplo#2	11796(26.6); 11120(27.6); **12135**(26.8); 11722(30.4); 9324(34.2)	11801(30.6); 12033(36.6); 11784(31.2); **12135**(33.8); CX97(31.0)	11979(34.4); **12135**(33.0); 11301(32.4); 11140(31.6); CX364(31.6)				11796(4.0); 11648(4.5); 11668(3.9); 11733(4.2); 10995(3.9)	12259(10.3); 11863(9.0); 11648(10.2); 11730(9.0); 11668(9.3)	11738(16.5); 11034(18.3); 10719(18.4); 10674(17.7); 10614(16.6)
		haplo#4				11820(63.1); **8066**(63.3); 12269(63.0); **8305**(71.8); 11953(62.7)	10609(80.8); **8066**(90.0); 11794(78.0); **8305**(87.0); 11953(89.2)	10609(103.4); **8066**(97.6); 11794(97.2); **8305**(101.1); 10503(98.2)			
		haplo#1				**10718**(70.6); B149(67.6); 11635(69.8); 12180(69.3); 9427(69.3)	11202(90.0); **10718**(105.2); 10719(88.6); 11663(89.4); B149(89.2)	**10718**(116.0); 8147(100.2); 11663(100.0); 10575(110.8); 12180(107.8); B011(100.0)			
*qSV3e*	*Os03g0799700*	haplo#3	10857(21.6); 11272(20.4); 10980(21.4); 10605(20.2); 11809(20.6)	11272(27.6); 11051(29.5); 10733(27.0); 11034(23.4); 10603(23.6)	10857(23.6); 11191(28.8); 11034(24.8); 10603(27.2); 11037(28.2)	10858(64.4); 10859(63.4); **10718**(70.6); **11663**(66.7); 9148(62.7)	10891(85.7); 10719(88.6); **10718**(105.2); **11663**(89.4); 9148(83.1)	10891(96.6); 10719(97.8); 10859(106.4); **10718**(116.0); **11663**(100.0)	10695(4.2); 11648(4.5); 11820(4.2); 11224(4.9); 10573(4.4)	12259(10.3); 11802(9.8); 11648(10.2); 10733(10.1); 10719(9.9)	10973(16.5); 11738(16.5); 10824(16.2); 11034(18.3); 10719(18.4)
		haplo#6	**12033**(17.4); **11863**(19.0); **11807**(20.0); 10695(21.6); 9023(17.0)	**12033**(36.6); **11863**(24.4); **11807**(25.2); 11182(21.2); 9023(24.6)	**12033**(29.2); **11863**(20.6); **11807**(28.8); 10942(21.0); 11140(31.6); B229(20.6)				11863(3.6); **11182**(3.5); **10973**(3.4); **10695**(4.2); B229(3.0)	11863(9.0); **11182**(8.4); **10973**(9.7); 11254(9.1); **10695**(8.2)	12033(15.8); 11807(12.7); **11182**(13.6); **10973**(16.5); **10695**(12.6)
		haplo#1	11805(28.2); **11453**(30.2); 11722(30.4); 9324(34.2); CX26(29.2)	11784(31.2); 12135(33.8); **11453**(33.0); 11273(33.2); CX26(32.8)	11979(34.4); 12135(33.0); **11453**(32.4); 11273(34.6); 11301(32.4)				11723(4.1); 11648(4.5); 11820(4.2); 11224(4.9); 10573(4.4)	11802(9.8); 11645(9.4); 11648(10.2); 11791(9.5); 10573(9.8)	11666(15.7); 11738(16.5); 11685(16.1); 10824(16.2); 8988(15.5)
		haplo#4	11799(19.0); 10954(16.2); CX2(17.4); CX73(19.4); **CX92**(16.2); CX114(17.6)	11799(25.6); 9590(21.0); CX2(21.2); CX84(20.4); **CX92**(25.0)	10954(30.6); 11351(30.4); 9590(22.0); CX53(18.6); **CX92**(20.2)						
	*Os03g0856700*	haplo#2	11624(23.2); 11622(26.4); 11763(24.8); 11733(23.2); 11260(23.2); 10986(23.2)	12033(36.6); 11784(31.2); 12135(33.8); 11453(33.0); 10898(34.4)	11979(34.4); 12135(33.0); 11453(32.4); 11140(31.6); CX364(31.6)	**10973**(68.4); 12180(69.3); B149(67.6); 8909(67.5); 8305(71.8)	11953(89.2); 10719(88.6); **10973**(89.2); B216(88.2); B149(89.2)	10609(103.4); **10973**(118.8); 12180(107.8); B011(100.0); 8305(101.1)	11648(4.5); 11733(4.2); 10986(4.1); 10614(4.1); 10695(4.2)	11693(10.3); 11802(9.8); 11648(10.2); 10719(9.9); 10733(10.1)	11738(16.5); 11034(18.3); 10719(18.4); 10614(16.6); 10973(16.5)
*qSV4c*	*Os04g0683600*	haplo#1	11805(28.2); **11453**(30.2); 11722(30.4); 9324(34.2); CX26(29.2)	12033(36.6); 12135(33.8); **11453**(33.0); 10898(34.4); 11273(33.2)	11979(34.4); 12135(33.0); **11453**(32.4); 11273(34.6); 11301(32.4)	**10718**(70.6); 11635(69.8); 12180(69.3); 9427(69.3); 8305(71.8)	11953(89.2); 10973(89.2); **10718**(105.2); 11663(89.4); B149(89.2)	10859(106.4); 10973(118.8); **10718**(116.0); 10575(110.8); 12180(107.8)	11648(4.5); 11820(4.2); 11224(4.9); 10573(4.4); 9427(4.5)	11693(10.3); 12259(10.3); 10733(10.1); 11648(10.2); 10719(9.9)	11738(16.5); 11034(18.3); 10719(18.4); 10674(17.7); 10614(16.6)
*qSV7c*	*Os07g0600400*	haplo#1	**11453**(30.2); 11722(30.4); B008(29.6); 9324(34.2); CX26(29.2)	12135(33.8); **11453**(33.0); 10898(34.4); 11273(33.2); B008(35.4)	11979(34.4); 12135(33.0); **11453**(32.4); 11202(37.8); 11273(34.6); 11301(32.4)	11635(69.8); 12180(69.3); 8967(68.7); 9427(69.3); 9964(68.5)	11953(89.2); 11202(90.0); 10973(89.2); 11663(89.4); B149(89.2); 9813(88.8)	10609(103.4); 10973(118.8); 10575(110.8); 11081(103.8); 12180(107.8)	11648(4.5); 11820(4.2); 10573(4.4); 10695(4.2); 9427(4.5)	11693(10.3); 12259(10.3); 10733(10.1); 11648(10.2); 10719(9.9)	11738(16.5); 10719(18.4); 10674(17.7); 10614(16.6); 10973(16.5)
		haplo#3	11057(16.0); 10605(20.2); **11034**(20.0); **11809**(20.6); 9403(22.6)	11191(22.6); **11034**(23.4); 11037(21.4); **11809**(22.8); 9403(22.8)	11062(27.6); 11191(28.8); **11034**(24.8); 11037(28.2); **11809**(23.4)	11671(60.8); 11289(58.1); **10718**(70.6); 9403(58.2); **8305**(71.8)	11671(78.3); 11324(79.9); 11034(77.6); **10718**(105.2); **8305**(87.0)	10871(93.8); 10602(93.6); 11034(95.4); **10718**(116.0); **8305**(101.1)	**11671**(3.3); **11034**(3.0); 10718(3.7); **B052**(3.4); 9403(3.7)	**11671**(8.7); **11034**(8.1); 10718(8.7); **B052**(8.2); 8305(8.1)	**11671**(15.2); 10602(15.0); **11034**(18.3); 10718(13.9); **B052**(13.8)

^a^TN = Tiller number, PH = Plant height, and DW = dry weight, respectively.

^b^A, B, C = Sampling on 27, 34, and 41 days after seeding, respectively.

^c^Donors were shown according to the sequencing code (http://www.rmbreeding.cn) except for the prefix of “IRIS_313-” were all omitted. Trait values of each donor was also shown in brackets. Donors behaved stably throughout three sampling stages were shown in bold and those carrying more than one elite haplotypes were also underlined.

## Discussion

### Comparison of identified SV-related QTL regions with reported genes/QTLs

Among the 42 SV-related QTL regions detected in this research, 18 (42.9%) were consistent with those of previous reports. Of these, 10 (*qSV1d*, *qSV2a*, *qSV2c*, *qSV3e*, *qSV4b*, *qSV5c*, *qSV6b*, *qSV7c*, *qSV8d*, and *qSV11a*) harboured more than one reported locus.

Among these QTLs, *qSV2c* was the most supported, and it was mapped with four reported loci, *qGR2* for germination rate^31^, *qSEV-2-2* for PH and plant dry weight^16^, *qCSH2* for seedling height, and *qSDW2* for SV-related traits under cold stress^32^. Another two QTL regions (*qSV1d* and *qSV3e*) were mapped with three reported loci. *qSV1d* was mapped with *qSSL1b* for seedling shoot length^33^, *qRL-1* for root length^34^, and *qLA-1* for leaf area^35^, while *qSV3e* was close to *qPHS3-2*/*OsGA20ox1* for seedling height^36^, *qSEV-3-4* for both PH and plant dry weight^16^, and *qSV-3-2* for shoot length and germination rate^13^. Seven QTL regions (*qSV2a*, *qSV4b*, *qSV5c*, *qSV6b*, *qSV7c*, *qSV8d*, and *qSV11a*) were each mapped with two reported loci. The *qSV2a* was mapped with *qFML2-1* for mesocotyl length^20^ and *qRL-2* for root length^35^; *qSV4b* was mapped with *qSDW4*.*2* for shoot dry weight^37^ and *qGP-4* for germination percentage^38^; *qSV5c* was mapped with *qFV-5-2* for seedling height^15^ and *qSEV-5-2* for both PH and plant dry weight^16^, although these two were detected in different tests of the same population. *qSV6b* was close to *qDW-6* for seedling dry weight^34^ and *qLDW-6* for leaf biomass^35^; *qSV7c* was mapped with *qSDW5-1* for shoot dry weight^11^ and *qSL-7* for shoot length^35^; *qSV8d* was mapped with *qSV-8-2* for germination rate, shoot length, and root length^13^ and with *qPR-8* for biomass portioning to roots^35^, while *qSV11a* was mapped with *qFML11-1* for mesocotyl length^20^ and with *qLN-11* for leaf number^35^. QTL regions for the SV-related traits that were identified in different mapping populations and diverse environments could be beneficial for the marker-assisted selection-based development of varieties with high levels of SV.

### Dynamic expression of SV-related QTL regions and their pleiotropy for SV-related traits

Improving SV has long been a focus of rice breeders. In particular, ESV (less than 28 DAS) is important for DSR under aerobic conditions in which they compete with weeds. In LSV, the tillering ability and biomass are the bases for the final yield components.

However, LSV is also based on the ESV. Thus, these two types of SV are highly associated. More than one third (42.9%) of the SV-related QTL regions detected in this work were mapped with reported ESV loci. However, four QTLs (*qSV1d*, *qSV2c*, *qSV3e*, and *qSV4b*) were not only mapped with more than one reported loci, but were also detected by both GAPIT and mrMLM, and all of them were responsible for PH. PH is an agronomic trait with a relatively high level of heritability. Thus, more QTL regions were responsible for PH (33 and 27 by GAPIT and mrMLM, respectively) compared with TN (26 and 23, respectively) and DW (30 and 23, respectively). In total, 11 of the loci at the QTL regions stably detected at all three sampling stages were responsible for PH. However, the number of stably detected QTL regions decreased to three and one for TN and DW traits, respectively.

One locus on chromosome 2 (*qSV2c*) affected all the three traits (TN, PH, and DW) throughout all three sampling stages (27, 34, and 41 DAS). This locus is also consistent with the three previously reported loci (*qGR2*^31^, *qCSH2*, and *qSDW2*)^32^. This stably expressed locus could be very useful and will be analysed further. However, the −LOG_10_(P) values for *qSV2c* were not very high. They ranged from 3.0 to 3.4, with a mean value of 3.2, for TN, from 3.8 to 4.7, with a mean value of 4.4, for PH, and from 3.0 to 3.3, with a mean value of 3.1, for DW.

The number of SV-related QTL regions expressed under different sampling stages varied according to the studied traits. For example, there were totally 28 loci responsible for TN, 33 loci for PH, and 31 for DW. The numbers of TN-related QTL regions for the three different sampling stages varied by 16, 11, and 16, respectively, while the numbers of PH-related QTL regions varied by 20, 26, and 21, respectively for the three stages. However, the numbers of DW-related QTL regions decreased by 18, 12, and 9, respectively. A dynamic pattern was determined for the SV-related QTL regions. In total, 33.3% of PH-related QTL regions were detected in all three sampling stages, and 36.4% were responsible for PH at two stages. Only 10.7% of TN-related and 3.2% of DW-related QTL regions were detected in all three stages, and 32.1% of the former and 19.4% of the latter were detected at two stages.

### Joint mapping of QTLs using GAPIT and mrMLM

GAPIT is based on a single dimension of genome scanning. We used the built-in maximum-likelihood method for the GWAS mapping of SV-related traits, while mrMLM was used because of its multi-locus nature. Thus, for trait variations controlled by multiple loci, the estimations of the loci effects by mrMLM should have greater confidence levels^24^.

Using a common setting, in comparison with GAPIT, mrMLM detected relatively fewer numbers of loci with a similar statistical power, indicated by −LOG_10_(P) values, in most cases. Additionally, as shown in Supplementary Figs 2–4, the threshold setting influenced QTL mapping. mrMLM focused on loci with high confidence levels and produced results with clearer backgrounds than GAPIT. This would be adequate for most molecular breeding or gene cloning applications. However, to determine the whole picture of a trait, a traditional package, such as GAPIT, should also be used. Conversely, as shown in this report, QTL regions can be located with GAPIT and then, supporting evidence obtained from mrMLM.

For the estimated allelic effects, of the 84 cases detected both by GAPIT and mrMLM, only 25 (29.8%) cases showed consistent effect directions. According to previous reports, with simulated data, a multi-loci method (mrMLM) is more powerful and more accurate in QTN effect estimation than single-locus methods^24^. All of these QTL regions were responsible for TN and PH rather than DW. Further experiments are required to clarify this inconsistency.

Additionally, as an example, we adopted SV mapping results from GAPIT with a suitable threshold setting to minimise possible type II errors. With the aid of mrMLM, the SV mapping results should have greater confidence levels. Thus, combining GAPIT and mrMLM is an option for our future GWAS mapping.

### Candidate gene identification in important QTL regions

In our 744 accessions, the average LD decayed significantly to 0.3 at a physical distance of ~50 kb (Supplementary Fig. 1). This is much less than the previously reported LD distance of 75 kb for *Xian*/*indica*^39^, not to mention the 150-kb LD distance for tropical *Geng*/*japonica*, and the >500-kb LD distance for temperate *Geng*/*japonica*. Thus, in this population, especially for the specific QTL regions, a sub-regional analysis with higher density markers may offer more useful information.

Based on the sub-region analysis using more SNPs and the haplotype analysis, five candidate genes, *Os01g0166800*, *OS03g0799700* and *Os03g0856700* (*OsGA20ox1*), *Os04g0683600*, and *Os07g0600400* were inferred for *qSV1a*, *qSV3e*, *qSV4c*, and *qSV7c*, respectively, using multi-evidenced QTNs. The candidate gene *Os01g0166800* for *qSV1a* is an E2F transcription factors target gene. *Os03g0799700* for *qSV3e* is a GTP1/OBG subdomain-containing protein. Both E2F- and GTP-related pathways are highly conserved in higher eukaryotes, and are involved in multiple basic functions, including cell cycle, DNA replication, and germination^40,41^. *Os03g0856700*, or *OsGA20ox1*, for *qSV3e* is a key gene responsible for seedling height, which is especially associated with ESV^36^, while later it is associated with spikelet number per panicle^42^. Thus, it is most likely the candidate gene for *qSV3e*. *Os04g0683600* for *qSV4c* is similar to the H0306F12.6 protein, which is a putative LRR receptor-like serine/threonine-protein kinase in maize but its function remains unclear in *Oryza*^43^. *Os07g0600400* for *qSV7c* codes a WD40/YVTN repeat-like domain-containing protein, which may function in the processes of signal transduction and stress adaptation^44^. The candidate genes for the four QTL regions need to be validated using transgenic or gene editing approaches in the future.

### Implications in the development of DSR cultivars

Both ESV and LSV are important for cultivars used in DSR cultivating systems. The ESV is highly correlated to the ability of rice seedlings to compete with weeds, especially under the aerobic condition (as in UDS)^45^. In PDS, weed control is much easier than in UDS. The plant type, which develops during the period of LSV, including the TN, PH, and DW contribute to the final grain yield. Most alleles at important stably detected QTL regions and/or in regions with multi-evidenced QTNs, such as *qSV1a*, *qSV3e*, *qSV4c*, and *qSV7c*, increase the TN, PH, and DW (Table 1). Further validation of favourable alleles at the above four stable QTL regions detected throughout the three sampling stages will be performed.

A rice cultivar with strong SV-related traits is desirable for direct seeding. However, SV-related traits have not been selected for in crop improvement programs focused on conventional breeding owing to their complex nature and quantitative inheritances. Molecular markers are effective in increasing selection efficiency, particularly for quantitative traits that are simply inherited. In this study, except for *qSV2c*’s roles in all three SV-related traits, there were 2 QTL regions (*qSV5a* and *qSV9b*) responsible for TN only, and 10 QTL regions (*qSV1c*, *qSV1d*, *qSV4a*, *qSV5c*, *qSV6a*, *qSV6b*, *qSV7a*, *qSV7c*, *qSV8c*, and *qSV10b*) responsible for PH only. A simple increase in PH would result in problems in the final maturation stage and may cause lodging, which is another key trait for DSR. A balance between TN and PH is favourable for increased yields. Thus, an appropriate combination of TN and PN for both a high SV at the seedling stage and a high yield potential at harvest could be achieved by pyramiding the QTLs underlying the two traits, such as *qSV5a* and *qSV9b* for TN and *qSV1c* and *qSV4a*for PH, after QTL validation using a marker-assisted selection approach.

With a high density of SNP markers, we identified 11 favourable haplotypes for four loci. These haplotypes, defined by key SNPs, can be transformed into breeder-friendly markers in the future. In particular, haplotype 2 of *qSV1a*, and haplotype 1, 4, and 6 of *qSV3e* offer more opportunities for the development of cultivars with greater biomasses, resulting from more tillers rather than taller plants. PDR cultivars with this kind of SV would not affect lodging resistance and would have relatively wider adaptations. In addition, elite donors for these favourable haplotypes were also identified (Table 3). For example, IRIS_313-12135 can be used as the donor for haplotype 2 of *qSV1a*, and IRIS_313-12033, IRIS_313-11863, and IRIS_313-11807 can be used for haplotype 6 of *qSV3e*. IRIS_313-11453 can be used as a donor for haplotype 1 of *qSV3e*, while CX92 can be the donor for haplotype 4 of *qSV3e*. For *OsGA20ox1*, IRIS_313-10973 could be a good donor for the elite haplotype 2.

## Conclusions

Tremendous variations for three SV-related traits, TN, PH, and DW, existed in the studied rice germplasms. Using a GWAS of GAPIT and mrMLM, 42 QTL regions, including 18 overlapping previously reported QTL regions and 24 new ones, for the SV-related traits were identified. Five candidate genes were inferred by fine-mapping using more SNPs and a haplotype analysis, including the known gene *OsGA20ox1* for *qSV3e*, which controls ESV and spikelet number per panicle at the maturing stage. Our results indicated that combining GAPIT and mrMLM is an option for GWAS mapping. Favourable alleles at stably expressed QTLs, such as *qSV1a*, *qSV3e*, *qSV4c*, and *qSV7c*, were mined, and their corresponding accessions were also identified. The results provide useful germplasms and genetic information for the future improvement of SV in rice.

## Supplementary information

Supplementary Information
