# Supplementary material for: Genetic dissection of seedling vigour in a diverse panel from the 3,000 Rice (Oryza sativa L.) Genome Project
Source: Sci Rep. 2019 Mar 18;9:4804. doi: 10.1038/s41598-019-41217-x (PMC6423299; doi:10.1038/s41598-019-41217-x)
Supplement: Supplementary file 1 — Supplementary Information [file 41598_2019_41217_MOESM1_ESM.pdf]

Genetic dissection of seedling vigour in a diverse panel from the 3,000 Rice (*Oryza sativa* L.) Genome Project

Kai Chen[1, 2, §], Qiang Zhang[3, §], Chun-Chao Wang[1], Zhi-Xia Liu[3], Yi-Jun Jiang[3], Lai-Yuan Zhai[1], Tian-Qing Zheng[1, *], Jian-Long Xu[1,2, *], Zhi-Kang Li[1,2]

[1] Institute of Crop Sciences/National Key Facility for Crop Gene Resources and Genetic Improvement, Chinese Academy of Agricultural Sciences, Beijing 100081, China.

[2] Agricultural Genomics Institute at Shenzhen, Chinese Academy of Agricultural Sciences, Shenzhen 518120, China.

[3] Institute of Rice Research, Guangdong Academy of Agricultural Sciences, Guangzhou 510640, China.

[§] These authors contributed equally to this work.

[*] Corresponding author. E-mail: xujlcaas@126.com, tonyztq@163.com

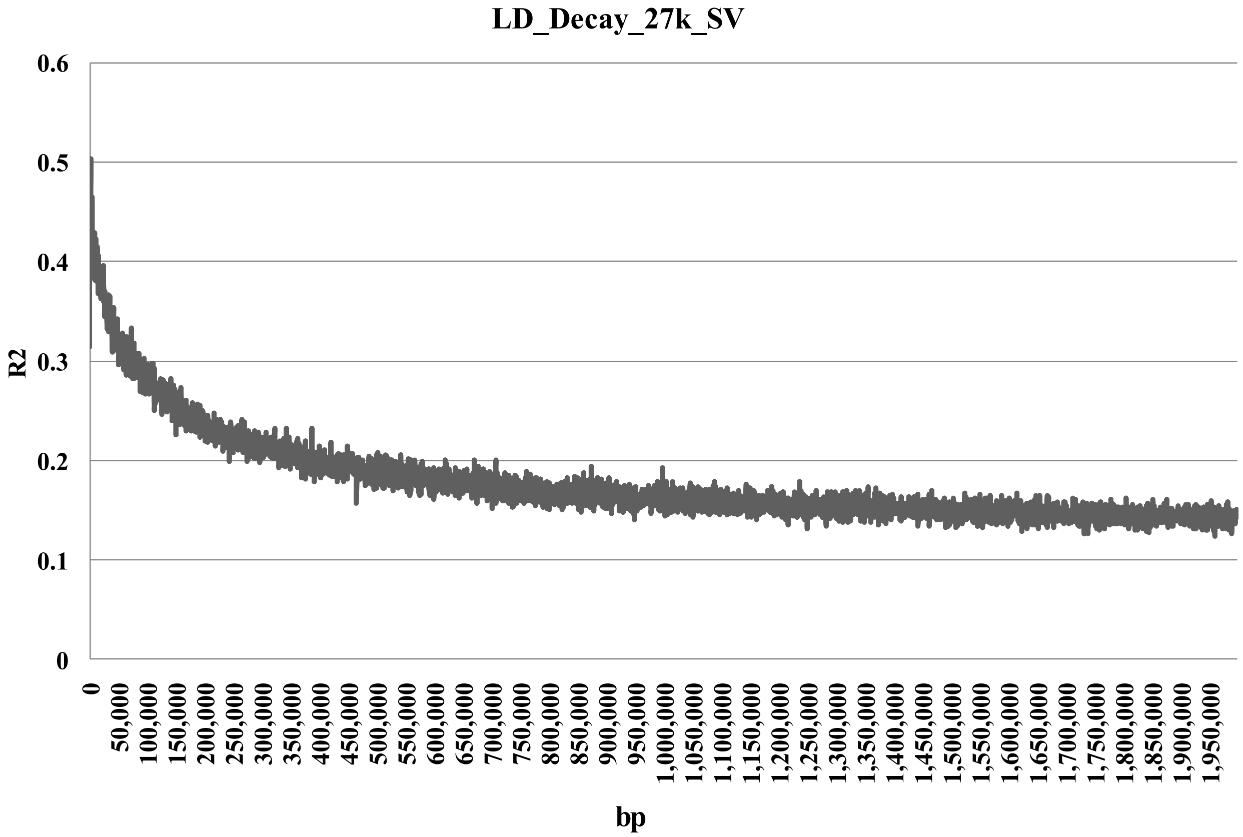

Supplementary Figure 1. LD decays throughout the genome in the 744 germplasm set.

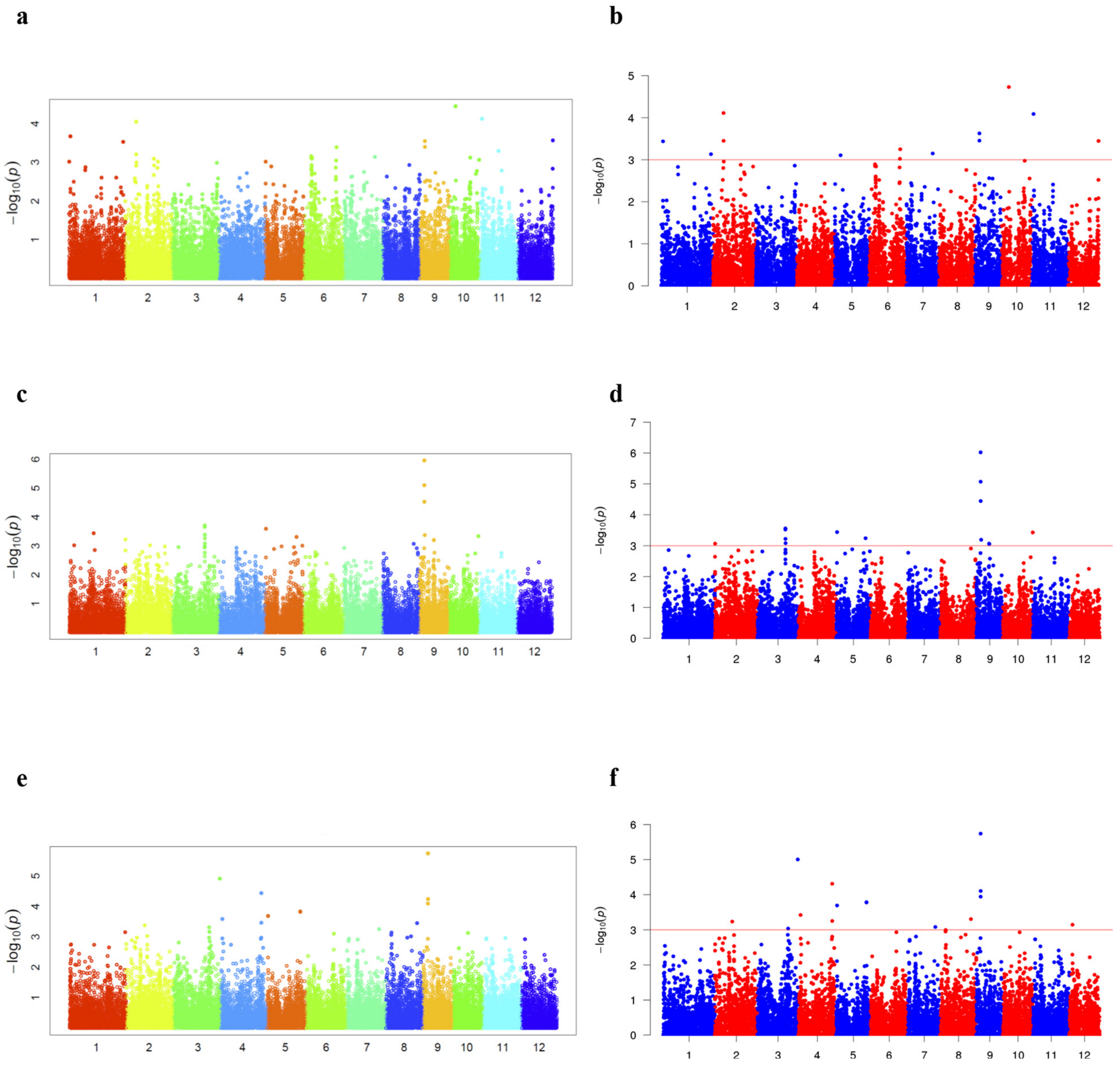

Supplementary Figure 2. Manhattan plots based on the GWAS results for tiller number (TN) of the 744 germplasms. **a**, **c**, and **e**: GWAS results by GAPIT for the sampling at 27, 34, and 41 days after seeding. **b**, **d**, and **f**: GWAS results by mrMLM for the sampling at 27, 34, and 41 days after seeding.

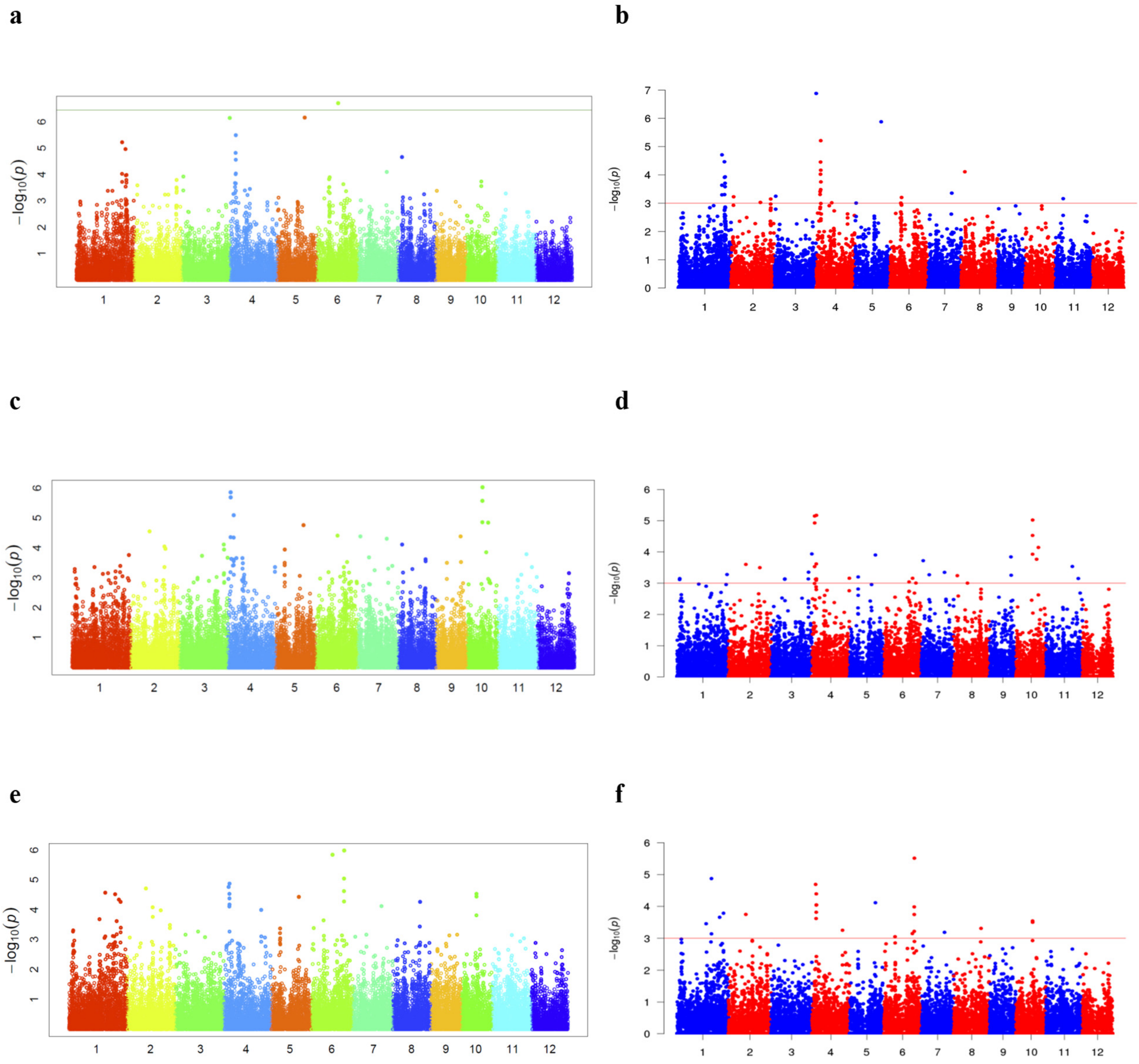

Supplementary Figure 3. Manhattan plots based on the GWAS results for plant height (PH) of the 744 germplasms. a, **c**, and **e**: GWAS results by GAPIT for the sampling at 27, 34, and 41 days after seeding. **b**, **d**, and **f**: GWAS results by mrMLM for the sampling at 27, 34, and 41 days after seeding.

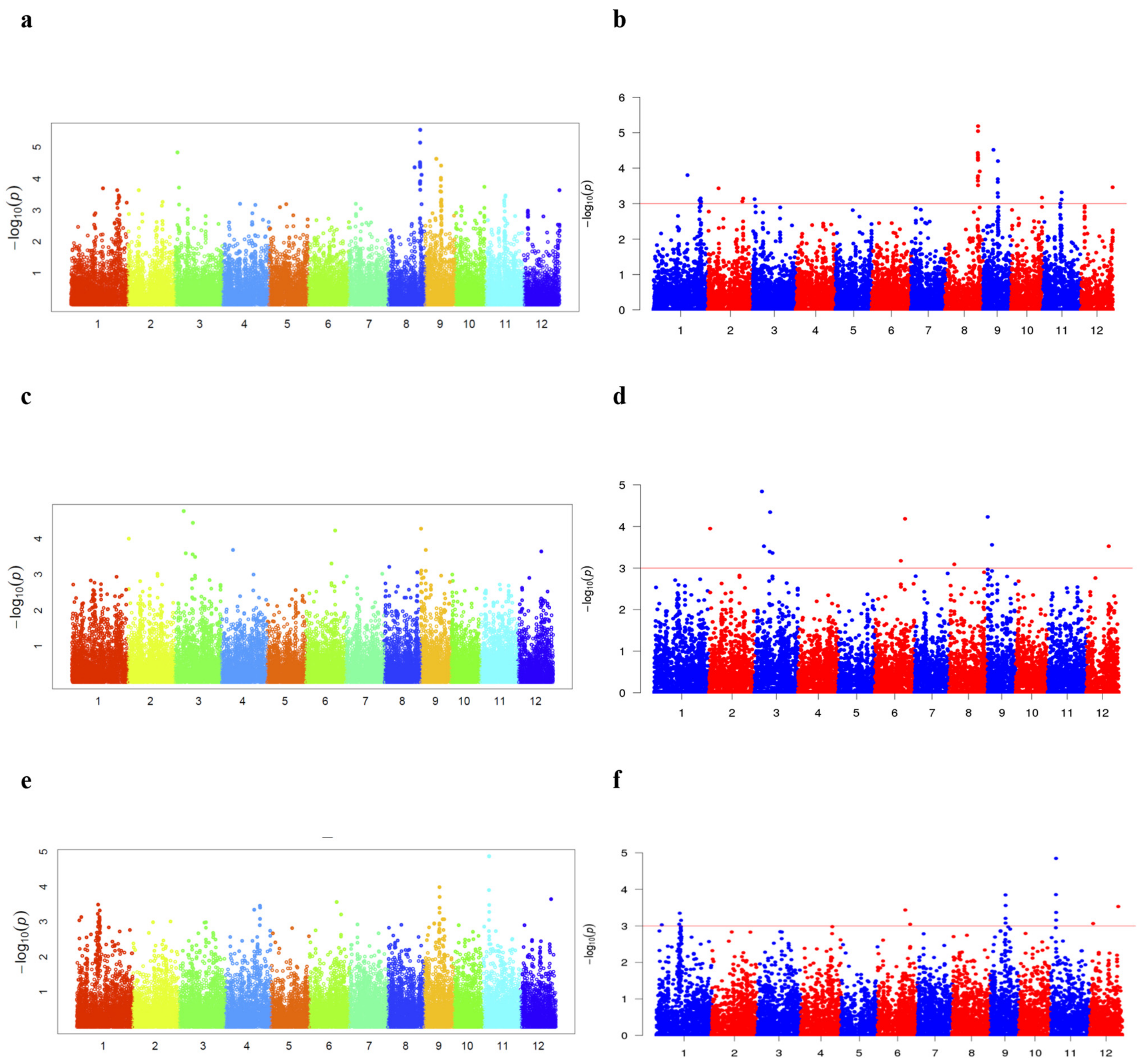

Supplementary Figure 4. Manhattan plots based on the GWAS results for dry weight (DW) of the 744 germplasms. a, **c**, and **e**: GWAS results by GAPIT for the sampling at 27, 34, and 41 days after seeding. **b**, **d**, and **f**: GWAS results by mrMLM for the sampling at 27, 34, and 41 days after seeding.

Supplementary Table. Significant SNP detected in the fine mapping for the multi-evidenced QTL regions affecting SV traits under three sampling stages.

QTL	SNP	Trait	Chr	Marker Pos (bp)	QTN effect	$-LOG_{10}(P)$	R^2 (%)
qSV1a	1_1472587	DW_B	1	1,472,587	-0.4	5.5	3.6
		DW_C	1	1,472,587	-0.6	6.9	4.0
	1_2020270	TN_A	1	2,020,270	-0.9	5.3	2.3
		DW_B	1	2,020,270	-0.2	4.7	1.4
	1_2236401	TN_B	1	2,236,401	-1.0	5.4	2.0
		TN_C	1	2,236,401	-1.2	8.0	2.8
	1_3117991	PH_A	1	3,117,991	1.3	7.3	2.0
		PH_B	1	3,117,991	1.5	5.6	1.4
	1_3209491	PH_B	1	3,209,491	-1.8	6.2	2.7
		DW_C	1	3,209,491	-0.3	4.3	1.2
	1_3218108	PH_A	1	3,218,108	-1.0	3.9	0.8
		PH_B	1	3,218,108	-1.2	4.5	0.6
	1_3426681	PH_A	1	3,426,681	1.0	5.1	1.6
		PH_B	1	3,426,681	1.3	6.0	1.3
		PH_C	1	3,426,681	2.4	12.3	3.5
qSV3e	3_33337125	PH_A	3	33,337,125	-0.9	5.7	1.1
		PH_C	3	33,337,125	-1.4	4.1	1.2
	3_36161416	PH_A	3	36,161,416	3.0	13.0	4.0
qSV4c	4_34907018	PH_A	4	34,907,018	2.1	8.6	1.9
		PH_B	4	34,907,018	2.3	7.3	1.2
		PH_C	4	34,907,018	2.6	7.6	0.8
qSV7c	7_21709213	TN_A	7	21,709,213	-1.6	8.5	3.8
		TN_B	7	21,709,213	-1.5	4.8	2.7
		DW_B	7	21,709,213	-0.5	5.5	3.3
	7_22156236	TN_A	7	22,156,236	-0.8	3.9	1.8
		TN_B	7	22,156,236	-1.2	5.1	2.6
	7_23973241	PH_A	7	23,973,241	-1.2	4.7	1.4
		PH_C	7	23,973,241	-2.8	6.2	2.8
	7_24497494	TN_A	7	24,497,494	1.3	7.0	3.7
		TN_B	7	24,497,494	1.3	6.5	3.0
